# Analysis and outcomes of wrong site thyroid surgery

**DOI:** 10.1186/s12893-021-01247-7

**Published:** 2021-06-04

**Authors:** Gianlorenzo Dionigi, Marco Raffaelli, Rocco Bellantone, Carmela De Crea, Carlo Enrico Ambrosini, Paolo Miccoli, Gabriele Materazzi, Antonio Ieni, Ettore Caruso, Daqi Zhang, Henning Dralle

**Affiliations:** 1 Division of Endocrine and Minimally Invasive Surgery, University of Messina, Messina, Italy; 2 U.O.C. Chirurgia Endocrina E Metabolica, Fondazione Policlinico Universitario A. Gemelli IRCCS, Roma, Italy; 3 Department of Surgery, University of Pisa, Pisa, Italy; 4 Division of Pathology, University of Messina, Messina, Italy; 5 Division of Thyroid Surgery, Jilin Provincial Key Laboratory of Surgical Translational Medicine, Jilin Provincial Precision Medicine Laboratory of Molecular Biology and Translational Medicine On Differentiated Thyroid Carcinoma, China-Japan Union Hospital of Jilin University, Changchun, 130000 China; 6 Sektion Endokrine Chirurgie, Klinik für Allgemein‑, Viszeral- und Transplantationschirurgie, Universitätsklinikum Essen, Hufelandstr. 55, 45147 Essen, Deutschland

**Keywords:** Wrong-site surgery, WSS, Malpractice, Medical errors, Patient safety, Thymectomy, Thymectomy instead of thyroidectomy, Wrong side, Wrong procedure, Wrong patient

## Abstract

**Background:**

In thyroid surgery, wrong-site surgery (WSS) is considered a rare event and seldom reported in the literature.

**Case presentation:**

This report presents 5 WSS cases following thyroid surgery in a 20-year period. We stratified the subtypes of WSS in wrong *target*, wrong *side*, wrong *procedure* and wrong *patient*. Only planned and elective thyroid surgeries present WSS cases. The interventions were performed in low-volume hospitals, and subsequently, the patients were referred to our centres. Four cases of wrong-target procedures (thymectomies [n = 3] and lymph node excision [n = 1] performed instead of thyroidectomies) and one case of wrong-side procedure were observed in this study. Two wrong target cases resulting additionally in wrong procedure were noted. Wrong patient cases were not detected in the review. Patients experienced benign, malignant, or suspicious pathology and underwent traditional surgery (no endoscopic or robotic surgery). 40% of WSS led to legal action against the surgeon or a monetary settlement.

**Conclusion:**

WSS is also observed in thyroid surgery. Considering that reports regarding the serious complications of WSS are not yet available, these complications should be discussed with the surgical community. Etiologic causes, outcomes, preventive strategies of WSS and expert opinion are presented.

**Supplementary Information:**

The online version contains supplementary material available at 10.1186/s12893-021-01247-7.

## Background

In the past 20 years, the American Institute of Medicine publication titled *To Err Is Human* identified wrong-site surgery (WSS) as a serious adverse effect in the healthcare system [1]. WSS is defined as surgery performed on the (1) wrong anatomical location with removing the wrong target (WTS, wrong target surgery), (2) wrong side (Wside), or wrong patient surgery (WPS), or (3) surgery by following a wrong, guideline discrepant procedure (GDS, guideline discrepant surgery) [2, 3]. WSS is also defined as a sentinel event, that is, an unexpected occurrence involving death or serious physical or psychological injures, or the risk thereof [4, 5]. Sentinel events require a root cause analysis, that is, a structured, systematic multidisciplinary approach to understand the processes involved in the WSS event [5].

Thyroidectomy is a type of surgery that may result in WSS [6]. Consequently, this specialty has few reports and institutional initiatives at preventing WSS [7, 8]. An open, mandatory process of reporting endocrine surgery incidents for relevant audit and awareness is necessary to prevent WSS [5].

This report presents 5 WSS cases following thyroid surgery.

## Case presentation

This case series used WSS data from the following 3 high-volume endocrine surgery centres in Italy: (1) Division for Endocrine and Minimally Invasive Surgery, University of Messina (about 500 endocrine procedures/year); (2) the Division for Endocrine Surgery, Fondazione Policlinico Universitario A. Gemelli, Università Cattolica del Sacro Cuore, Rome (3.500/year); (3) Division of Endocrine Surgery, University of Pisa (3.500/year). This analysis included patients undergoing thyroid surgery between 2000 and 2019. In this series review, WSS cases following parathyroid and adrenal, but also neuroendocrine tumor (NET)-gut and NET-pancreas surgery were not included.

All subjects gave their informed consent for inclusion before they participated in the study. The study was conducted in accordance with the Declaration of Helsinki.

This study was exempted from ethical review by the Research Ethics Committee.

All reports were carefully reviewed to identify the cause of the error based on definitions established by the Joint Commission (Additional file 1: Table S1) [9]. WSS is defined in Additional file 2: Table S2.WTS, Wside, WPS, GDS were the primary outcomes described. Additionally, we offer an expert opinion from Surgeon who is the senior author of the present article (HD). The average number of WSS cases per year was calculated and analysed using statistics software (Statistical Package for the Social Sciences Inc., Chicago, IL). Calculation of WSS cases: calculation means numbers of WSS cases per total numbers of (thyroid) cases operated on during the time period analyzed.

## Discussion and conclusion

The main objective was to describe the incidence of WSS and highlight the risk factors that can help limit the possible occurrence of complications or adverse events in thyroid surgery (Additional file 7: Fig. S3). These WSS cases are considered the first actual cases observed following thyroid surgery. This topic of thyroid malpractice surgery at the wrong site is greatly underestimated in the literature and in congresses alike.

### Contingent definitions

The act of defining wrong *target*, *side*, and *patient* thyroid surgery is clear, allowing to describe and compare errors conditions [10–13]. However, it is more difficult to define a wrong *procedure,* i.e., GDS considering also that procedural recommendations from societies’ guidelines are constantly evolving and refining [14]. Nevertheless, we believe that 2 cases described in this study represent the possibility of a dual WSS, both of wrong *target* (thymectomy instead of thyroidectomy) and *procedural* error (lymphadenectomy of the central compartment has not been performed or completed).

### Prevalence

The question arises how often thyroid WSS are occurring at the National level. Our data show that there exists a non-zero incidence of WSS in thyroid surgery [9]. In general surgery, the estimated range of WSS varies widely, ranging from 0.09 per 10,000 to 4.5 per 10,000 procedures [10, 15, 16]. The total number of endocrine surgeries performed during the observation within our three high centers were worthwhile mentioned in the methods section: thus, the prevalence was 0.0035 (i.e., 5 over about 140.000 in 20 year period). However this does not represent the real prevalence of WSS, because we are the centers that have reoperated WSS cases. We are not aware of the true numbers of the centers where the surgical error occurred. We know with certainty that they are not centers specialized in thyroid surgery. We recommend that every thyroid surgeon remains cognizant of the fact that WSS thyroid excision may be observed. Endocrine surgery database may initiate programmes for the mandatory reporting of WSS events for tracking and quality improvement purposes in all hospitals regardless of accreditation.

### Specialities

Our report showed that the incidence of WSS reported by general; ear, nose, and throat (ENT); and endocrine surgeons is relatively similar. Recent survey-based studies demonstrated that medical errors and adverse events are commonly and consistently observed in general surgery and otolaryngology [4, 5].

### Causes

During incision, the neck presents a unique challenge in that a single inaccurate surgical incision (significantly high or low) or a remote incision yields access to multiple potential surgical sites. Mistaken identification of structures in the neck is a potentially troublesome phenomenon. Paying careful attention to one of the patients experiencing WSS in this study, the patient with a long neck, is considered beneficial. Most patients in this study had small thyroid glands and nodules/tumours (<10 mm).

None of the patients in this study underwent endoscopic or robotic procedure. A procedure (representative case [n = 2]) was initially performed using a 3-cm mini-incision. Considering the rarity of the event, conclusions as to whether endoscopic or robotic surgery can be excluded from this WSS cannot be established.

WSS presented in this series were performed by surgeons with mean age of 41 years (range 27–55 years). The surgeon in the case series (#2) was a resident in general surgery, while the other surgeons were low-volume both general (n = 2) or ENT surgeons (n = 2). The surgeons involved do not perform more than 10-20 thyroidectomies per year (Table 1). Thus, the incidence of WSS as a result of inexperienced surgeons should be ruled out.

**Table 1:** WSS cases overview (N = 5)

Case No.	Year	Speciality type	Age/Gender Surgeon	Thyroid cases operated/year	Age/Gender Patient	Community	Employed	Indication of surgery	Anatomical dominant side	Dominant nodule size (mm)	Gland volume (mL)	Planned procedure	Type of surgery	E/R/Min/O	Use of MG	Use of FS	Use of IONM	Organ excised	Surgery duration (min)	No. of surgeons ^	Mentor in OR	Pathology	Error	Malpractice claim
1	2017	General surgery	27/M	10	35/F	Sinhalese	No	Bethesda III	Right	10	21	Right lobectomy	Left lobectomy	M	No	No	No	Thyroid	20	3	No	Malignant	Wrong side	No
2	2019	Head & Neck	33/F	10	42/F	Hispanidad	Yes	PTC with LN metastases	Left	6	19	Thyroidectomy + central + left lateral node dissection	Thymectomy + left lateral lymph node dissection	O	Not known	No	No	Thymus + LN	130	2	Not known	Malignant	Wrong target Wrong procedure	Yes
3	2014	Head and neck	60/M	20	20/F	Caucasian	yes	PTC	right	8	5	Thyroidectomy	Paratracheal lymphnodes removal	O	No	No	No	Lymph nodes	130	2	Not known	Malignant	Wrong target	Yes
4	2002	General Surgery	52/M	Not known	41/F	Caucasian	Yes	Toxic goiter	Bilateral	30	34	Total thyroidectomy	Removal of thymic tissue	O	No	No	No	Thymic tissue	60	3	No	Benign	Wrong target	No
5	2019	General Surgery	55/M	10	17/F	Caucasian	No	Bethesda V	Right	15	23	Total Thyroidectomy	Removal of thymic tissue + central LN	O	No	No	No	Thymic tissue + central LN	80	2	No	Malignant	Wrong target Wrong procedure	No

*F* female, *M* male, *E* endoscopic, *R* robotic, *Min* minincision (< 3 cm), *O* open surgery, *MG* magnify glasses, *FS* frozen section, *IONM* intraoperative neural monitoring, *^ No. of surgeons* surgeons in the operating room, *OR* operative room, *LN* lymph nodes

According to our data, the most common cause of WSS was human error, comprising approximately 80% of cases (Additional file 3: Table S3). Failure of leadership was also identified by the Joint Commission to be the most common cause of WSS nationally [9]. Leadership and human error etiologic causes are defined by the Joint Commission as a lack of organisational planning, resulting in noncompliance or ineffective implementation of timeout procedures, which include marking the incorrect site, failure to mark any site, or markings being obscured by surgical drapes [9].

### Clinical effects of WSS

All 5 patients in this study required revision surgery. The sequelae between wrong-*target versus* wrong-*side* surgery are slightly different (Additional file 4: Table S4). Participation in WSS may negatively influence the perception of safety of healthcare professionals in the operating room [17].

### Preventive strategies

We recommend that every thyroid surgeon should develop an algorithm to confirm, in every surgical case, that he or she is not excising the wrong thyroid lobe or organ. The proper implementation of timeout protocols requires a concerted effort by administrators and clinicians to follow and complete the checklists also for thyroid surgery. To eliminate wrong-site, wrong-side, and wrong-procedure surgery, a preoperative verification process to confirm documents and to implement a process to mark the surgical site (Additional file 6: Fig. S2) and involve the patient/family should be performed [9].

We further suggest that the surgeon should develop an adequate exposure. Deliberate identification of known landmarks was identified as the most useful strategy in preventing wrong excision [9]. Intraoperatively, once the surgeon creates a Kocher incision, the correct structures must be identified.

However, the present series emphasises the importance of adequate training and experience (i.e., annual case volume and cumulative experience) for surgeons dealing with thyroid surgery [10, 18, 19]. All the reported WSS cases would have been prevented if the primary surgery has been performed and/or supervised by an experienced endocrine/thyroid surgeon.

The role of routine use of magnifying glasses (MG), intraoperative frozen section (FS) and intraoperative neural monitoring (IONM) should be emphasized. While it is conceivable that a surgeon removes the contralateral lobe (not informed about preoperative findings), it is unacceptable (serious mistake) to remove thymic tissue instead of thyroid. IONM, MG and FS cannot avoid removing the wrong side, but the wrong tissue (Additional file 5: Fig. S1a–d). The “wrong location/wrong target surgeries” have not used IONM for anatomic identification of structures (e.g., crossing point of ITA and RLN with clear relationship to posterior thyroid capsule), MG, or FS for diagnosis of tissue removed.

### Grade of severity of surgical error and legal action

Case by case review may classify the grade of severity of surgical error as to one of three grades of severity of surgical error:

- (+ + +) highest grade of severity: this serious surgical error simply is not understandable, absolutely unacceptable and refers for instance to wrong location/wrong target removing surgery (e.g., thymic tissue instead of thyroid tissue);
- (+ +) second grade of severity: wrong side thyroid surgery. This surgical error is a deficit of communication between diagnostic performing doctor (general practitioner/endocrinologist/nuclear medicine) with operating surgeon, or vice versa, or simply the error of the surgeon who did not read exactly the preoperative findings or not concentrate during surgery;
- (+) lowest grade of severity: wrong, guideline disconform thyroid surgery. Lowest grade of severity, because interpretation and severity grading very much depends on the individual case, the individual description within the operative report, the individual disease, intraoperative findings and decision making, outcome of disease, and operative complications.

This surgical classification of severity of (surgical) errors do not completely overlap with legal interpretation, because legal interpretation mainly would ask for the disadvantage of the patient having had two surgeries (summing up initial unnecessary surgery without/with complications).

Dralle et al. reported 1 case of operation on the wrong side, with over 75 verdicts on malpractice claims after thyroid surgery [6]. However, according to Abadin SS et al., WSS cases resulting in thyroid surgery malpractice were not described [7]. In our series, 40% (2/5) of WSS led to legal action against the surgeon or a monetary settlement [6].

### Expert comment (*HD*)

I do not remember any WSS case at my departments at Hannover Medical School, Halle, and Essen University Hospital, however, we did not document the term “WSS” in our documentation system. I have drawn up 1995 through 2019 in total 283 expert reviews of general/visceral surgery medicolegal cases commissioned by state courts or arbitration boards for medical liability issues related to surgeries in various hospitals (not my own departments). 188/283 (66.4%) have been thyroid surgery cases. Only one out of 188 thyroid surgery malpractice cases (0.5%) has been WSS surgery: a 59 year old female with completion thyroidectomy at the wrong side (right side reoperated instead of removal of left thyroid remnant after right hemithyroidectomy and left subtotal resection due to papillary thyroid cancer). The year of malpractice review was 2003. In contrast to the only one WSS case of my malpractice expert review series 3/188 (1.6%) thyroid surgery malpractice cases dealt with overlooked, not removed target nodules in the thyroid. During the period 1995 through 2019, 3 cases were reviewed: two cases in 1996 and one case in 2003.

After 2003 I had no additional malpractice expert review case with either WSS issue, or overlooked thyroid nodule. In summary, concerning my own malpractice expert review series for medicolegal thyroid surgery cases (a) WSS cases were extremely rare (1/188; 0.5%), overlooked/forgotten thyroid target nodules more frequent (3/188; 1.6%), however, all 4 cases (4/188; 2.1%) in my series were before 2004. The 3 cases with overlooked/not removed thyroid target nodules were all thyroid surgeries with subtotal resection.

## Limitations of the report

First, the number of thyroid WSS was low (5 cases); hence, concerns about the true prevalence of WSS arise. Furthermore, the reader understands that creating a discussion with these limited cases presented is considered difficult. Second, this study involved 3 centres; thus, it was not designed to represent all thyroid surgeons. Consequently, its findings cannot be generalised. Third, because of the retrospective design of this study, we cannot determine the precise causality of WSS. Fourth, this study may be affected by information bias. WSS is a sensitive topic, and surgeons may have underreported their errors. Further studies will be required to conduct a survey among all professions working in the operating room (OR), such as scrub nurses, anaesthetist nurses, auxiliary nurses, or other healthcare professionals involved in OR safety. Fifth, the data collection of this study was conducted between 2000 and 2019. Considering evolving trends in the safety culture of OR, the relevance of these findings needs to be updated.

This review has identified 5 planned thyroid surgical procedures associated with WSS. All 5 patients presented were mistakenly operated at other healthcare facilities (1 in South America, 4 in Italy) and subsequently referred to our centres (Table 1). The patients reported comprised 1 Hispanidad, 1 Sinhalese, and 3 Italian subjects. The median age of the patients was 31 years (range 17–42 years). All the patients were female, and 3 of the 5 patients were employed. The mean dominant nodule size was 13 mm (range 6–30 mm). The mean thyroid gland volume was 20 mL. All initial surgical procedures were performed open and conventionally, mostly for the treatment of malignancy (80%). The most common etiologic cause identified was human error (Additional file 3: Table S3). The most common error was WTS (4/5), followed by GDS (n = 2) and Wside (n = 1). WPS was not detected in the review. Reported events were stratified by surgical specialty and anatomical site (Table 1). The study shows that only planned and elective thyroid surgeries present WSS cases. WSS presented in this series were performed by surgeons with mean age of 41 years (range 27–60 years). The surgeon in the case series (#2) was a resident in general surgery, while the other surgeons were low-volume both general (n = 2) or ENT surgeons (n = 2). A Additional file 8 offers the description of the 5 references cases.

## Supplementary Information

**Additional file 1: Table S1.** Definitions for WSS * [9].

**Additional file 2: Table S2.** Definitions for wrong endocrine surgery. Some procedures may include more than one type of error.

**Additional file 3: Table S3.** Causes of WSS. Some procedures can include more than one type of error.

**Additional file 4: Table S4.** Consequences may be different between surgical errors. Indications are the same as for.

**Additional file 5: Figure S1.** a–d (a) Normal thymic tissue within the cortex, mainly comprising lymphocytes and medulla with epithelial component. (b) In encapsulated invasive papillary thyroid carcinoma, follicular variant, low magnification revealed neoplastic proliferation that showed a predominantly follicular growth pattern surrounded by a fibrous capsule (x10, haematoxylin/eosin stain). (c) At higher magnification, the tumour was characterised by elongated follicles with fibrohyaline band formation, nuclear features reminiscent of papillary thyroid carcinoma, and luminal colloid with scalloped edges (x20, haematoxylin/eosin stain). (d) Lymph node with metastatic deposit (x20, haematoxylin/eosin stain).

**Additional file 6: Figure S2.** Preoperative radiological image showing thyroid gland still in the anatomical site.

**Additional file 7: Figure S3.** Possible risk factors and preventive strategies for wrong-site surgery with emphasis given to thyroid surgery.

**Additional file 8.** References cases description.

## Data Availability

Not applicable.

## Abbreviations

RLN: Recurrent Laringeal nerve
IONM: Intraoperative neural monitoring
FS: Frozen section
MG: Magnifying glasses
ENT: Ear, nose, and throat
NET: Neuroendocrine tumor
WSS: Wrong-site surgery
WTS: Wrong target surgery
Wside: Wrong side
WPS: Wrong patient surgery
GDS: Guideline disconform surgery
