# Supplementary material for: Analysis and outcomes of wrong site thyroid surgery
Source: BMC Surg. 2021 Jun 4;21:281. doi: 10.1186/s12893-021-01247-7 (PMC8176686; doi:10.1186/s12893-021-01247-7)
Supplement: Supplementary file 1 — Additional file 1: Table S1. Definitions for WSS * [9]. [file 12893_2021_1247_MOESM1_ESM.docx]

**Supplementary Table 1.** Definitions for WSS * (9)

| **Cause** | **Definition** |
| --- | --- |
| Leadership | Organizational planning/culture, community relations, service availability, priority setting, resource allocation, complaint resolution, leadership collaboration, standardization (eg, clinical practice guidelines), directing department/services, integration of services, inadequate policies and procedures, noncompliance with policies and procedures, performance improvement, medical staff organization, nursing leadership. |
| Human factors | Staffing levels, staffing skill mix, staff orientation, in-service education, competency assessment, staff supervision, resident supervision, medical staff credentialing/privileging, medical staff peer review, other (eg, rushing, fatigue, distraction, complacency, bias) |
| Communication | Oral, written, electronic, among staff, with/among physicians, with administration, with patient or family |
| Assessment | Adequacy, timing, or scope of assessment; pediatric, psychiatric, alcohol/drug, and/or abuse/neglect assessments; patient observation; clinical laboratory testing; care decisions |
| Information management | Information management needs assessment, confidentiality, security of information, data definitions, availability of information, technical systems, patient identification, medical records, aggregation of data |
| Operative care | Operative care planning, blood use, and/or patient monitoring |
| Physical environment | General safety, fire safety, security systems, hazardous materials, emergency management, smoking management, equipment management, utilities management |
| Patient rights | Informed consent, participation in care, end-of-life care, pain management, privacy |
| Anesthesia care | Planning, monitoring, and/or discharge |
| Continuum of care | Access to care, setting of care, continuity of care, transfer of patient, and/or discharge of patient |

* Table derived from Reference n.9
