# Supplementary material for: Analysis and outcomes of wrong site thyroid surgery
Source: BMC Surg. 2021 Jun 4;21:281. doi: 10.1186/s12893-021-01247-7 (PMC8176686; doi:10.1186/s12893-021-01247-7)
Supplement: Supplementary file 2 — Additional file 2: Table S2. Definitions for wrong endocrine surgery. Some procedures may include more than one type of error. [file 12893_2021_1247_MOESM2_ESM.docx]

**Supplementary Table 2.** Definitions for wrong endocrine surgery. Some procedures may include more than one type of error

|  | Definition | Example  Planned Surgery | Example  Error |
| --- | --- | --- | --- |
| Wrong *target surgery (WTS)* | Procedure performed on an incorrect location, incorrect anatomical site removing the wrong target | Thyroidectomy | Thymectomy |
| Wrong *side (WSS)* | Procedure performed on the contralateral side | Thyroid: left lobectomy for toxic adenoma | Thyroid: right lobectomy |
| Wrong *patient* surgery (WPS) | Surgery intended for one patient to another patient |  |  |
| Guideline disconform procedure (GDS) | Incorrect or additional procedure that was conducted | Patient with thyroid cancer and bulky lymph node metastases | Lymph nodes not removed |
