# Supplementary material for: Analysis and outcomes of wrong site thyroid surgery
Source: BMC Surg. 2021 Jun 4;21:281. doi: 10.1186/s12893-021-01247-7 (PMC8176686; doi:10.1186/s12893-021-01247-7)
Supplement: Supplementary file 3 — Additional file 3: Table S3. Causes of WSS. Some procedures can include more than one type of error. [file 12893_2021_1247_MOESM3_ESM.docx]

**Supplementary Table 3.** Causes of WSS. Some procedures can include more than one type of error

| **Cause** | **n** |
| --- | --- |
| Leadership | 2 |
| Human factors | 4 |
| Assessment | 1 |
| Communication | 1 |
| Operative care | 2 |
| Information management | 0 |
