# Supplementary material for: Analysis and outcomes of wrong site thyroid surgery
Source: BMC Surg. 2021 Jun 4;21:281. doi: 10.1186/s12893-021-01247-7 (PMC8176686; doi:10.1186/s12893-021-01247-7)
Supplement: Supplementary file 4 — Additional file 4: Table S4. Consequences may be different between surgical errors. Indications are the same as for. [file 12893_2021_1247_MOESM4_ESM.docx]

**Supplementary Table 4.** Consequences may be different between surgical errors. Indications are the same as for

|  | **Consequences** |
| --- | --- |
| **Wrong *anatomical site* and *procedure***  **(Case 1)** | ***Preoperative***   - Internal audit - Review previous medical/imaging/surgical/pathological documentation - Enhance imaging (CT scan) to verify any neck abnormal or distorted anatomy - Confirm wrong site/procedure surgery - Laryngeal examination to verify both RLNs function - Refer to specialized endocrine surgery center - Verify coexisting parathyroid disease - Patient counseling and consent   ***Intraoperative strategy***   - Re-schedule surgery - Increase morbidity - Challenge of proper redo-surgery timing - Expect adhesions from previous surgery - Choose open surgery access - Pick first operative case of morning - Lateral "back door" approach - Increased risk of RLN injury - Use of nerve monitoring - Frozen section may be of value   ***Postoperative implications***   - Increased risk of transient/permanent hypocalcemia - Malpractice action - Patient distress - Financial |
| **Wrong *side***  **(Case 2)** | ***Preoperative***   - Internal audit - Review previous medical/imaging/surgical/pathological documentation - Employ imaging to confirm laterality (US) - Confirm wrong side surgery - Schedule laryngeal examination to verify first side RLN function - Patient counselling and consent   ***Intraoperative strategy***   - Re-set surgery   ***Postoperative*** ***implications***   - Increased risk of transient/permanent hypocalcemia - Malpractice action - Patient distress - Financial |

initial exploration.

RLN: recurrent laryngeal nerve

CT: computed tomography

US: ultrasonography
