# Supplementary figures and images for: Analysis and outcomes of wrong site thyroid surgery
Source: BMC Surg. 2021 Jun 4;21:281. doi: 10.1186/s12893-021-01247-7 (PMC8176686; doi:10.1186/s12893-021-01247-7)

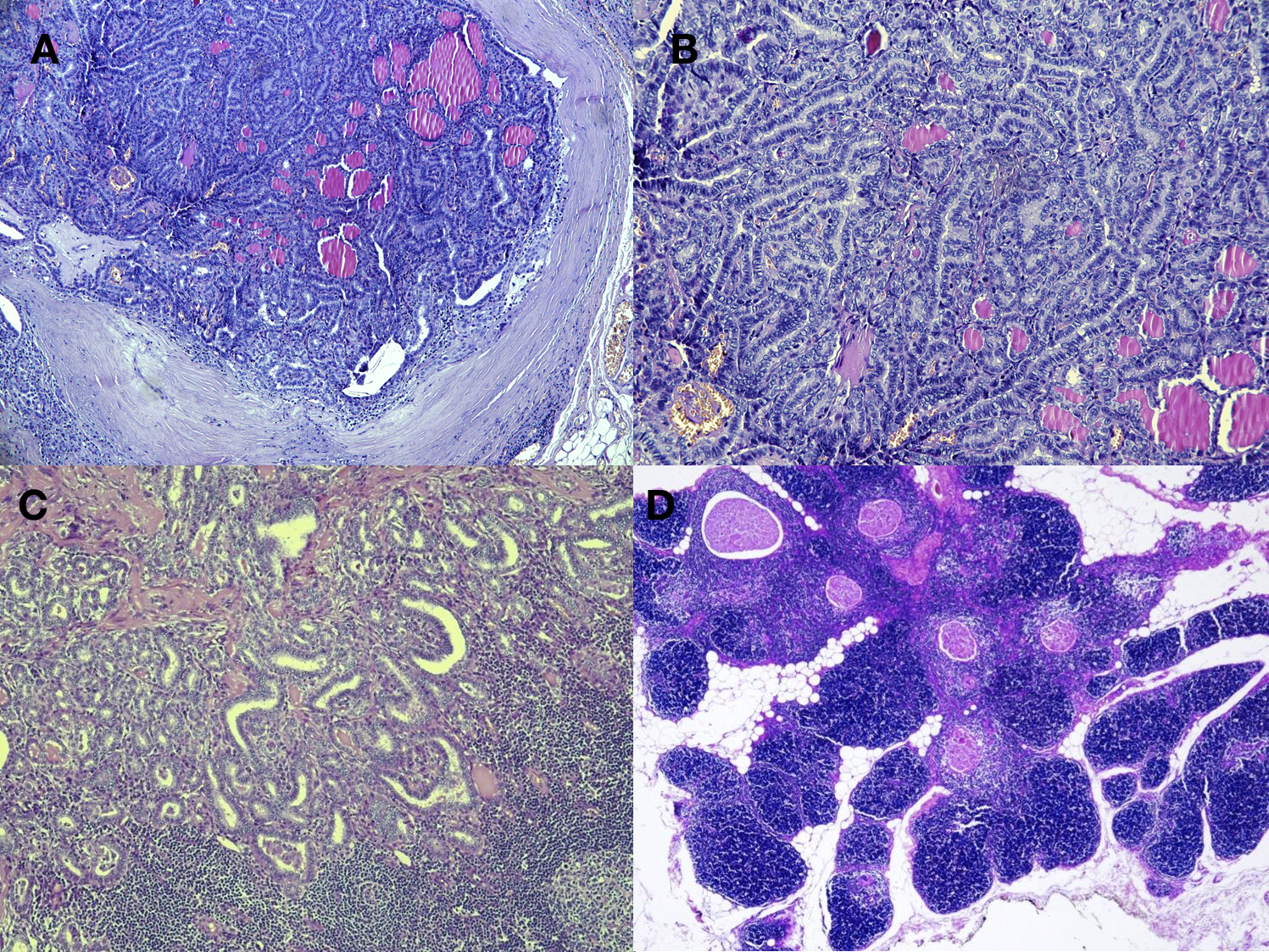

Supplement: Supplementary file 5 — Additional file 5: Figure S1. a–d (a) Normal thymic tissue within the cortex, mainly comprising lymphocytes and medulla with epithelial component. (b) In encapsulated invasive papillary thyroid carcinoma, follicular variant, low magnification revealed neoplastic proliferation that showed a predominantly follicular growth pattern surrounded by a fibrous capsule (x10, haematoxylin/eosin stain). (c) At higher magnification, the tumour was characterised by elongated follicles with fibrohyaline band formation, nuclear features reminiscent of papillary thyroid carcinoma, and luminal colloid with scalloped edges (x20, haematoxylin/eosin stain). (d) Lymph node with metastatic deposit (x20, haematoxylin/eosin stain). [file 12893_2021_1247_MOESM5_ESM.tiff]

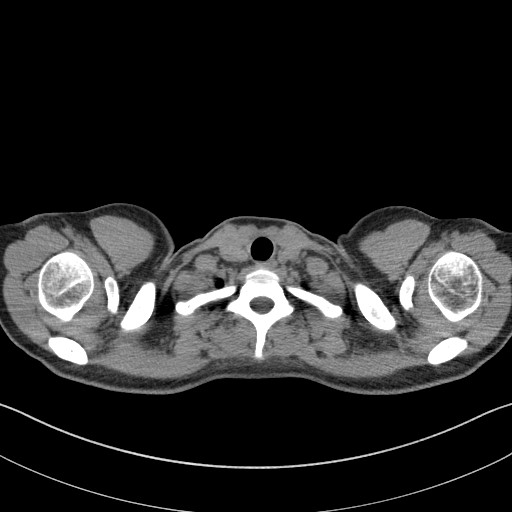

Supplement: Supplementary file 6 — Additional file 6: Figure S2. Preoperative radiological image showing thyroid gland still in the anatomical site. [file 12893_2021_1247_MOESM6_ESM.tiff]

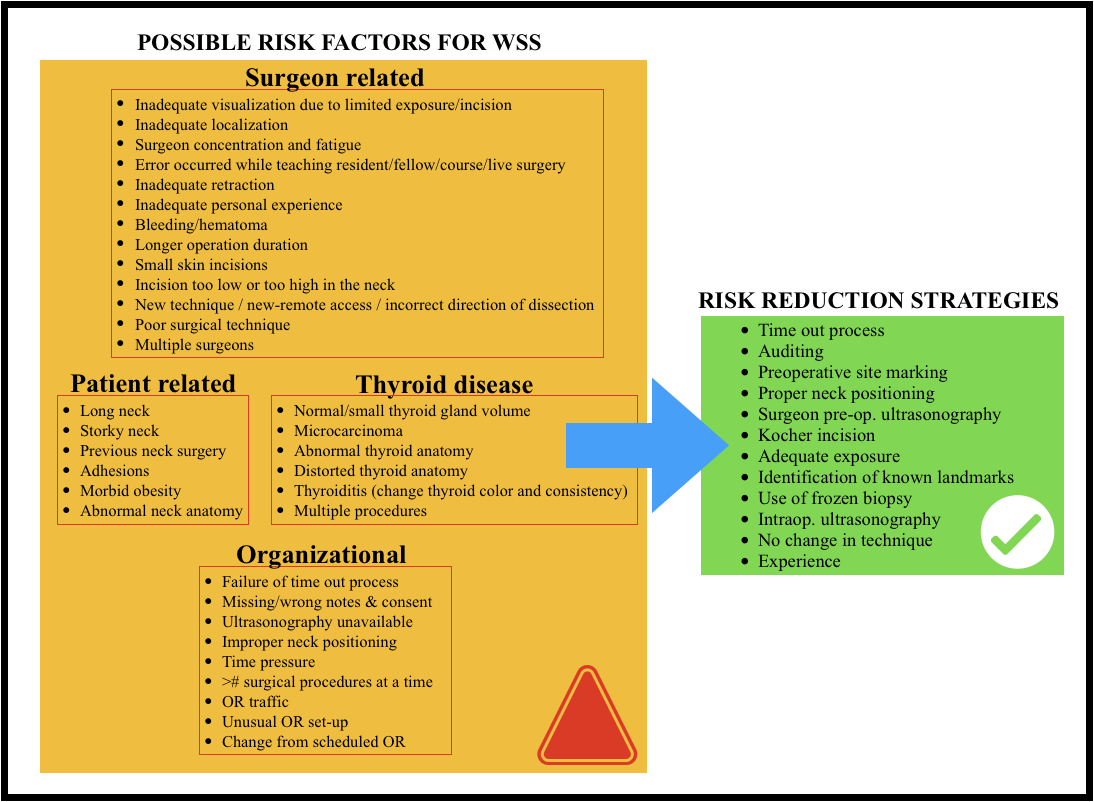

Supplement: Supplementary file 7 — Additional file 7: Figure S3. Possible risk factors and preventive strategies for wrong-site surgery with emphasis given to thyroid surgery. [file 12893_2021_1247_MOESM7_ESM.tiff]
