# Supplementary material for: Analysis and outcomes of wrong site thyroid surgery
Source: BMC Surg. 2021 Jun 4;21:281. doi: 10.1186/s12893-021-01247-7 (PMC8176686; doi:10.1186/s12893-021-01247-7)
Supplement: Supplementary file 8 — Additional file 8. References cases description. [file 12893_2021_1247_MOESM8_ESM.docx]

**REFERENCES CASES DESCRIPTION**

**Reference Case 1: thymectomy instead of thyroidectomy**

A 39-year-old Hispanidad woman underwent ultrasound (US) examination and subsequently a US-guided fine-needle aspiration (US-FNA) in the neck on January 2019 in Mexico. US revealed a nodule (4 × 6 mm) in the left lobe of the thyroid and a nodule (4 × 4 mm) in the right lobe and several swollen lymph nodes in the left side of the neck (~2–3 cm). US-FNA cytology of one lateral left neck lymph node revealed metastatic papillary thyroid carcinoma (PTC). On February 2019, the patient underwent surgery in South America. Definitive histological examination described 4/14 metastatic PTC lymph nodes (left lateral compartment) and normal thymic tissue (Supplementary Figure 1). Thyroid tissue was not observed on permanent pathology. Hence, the patient scheduled an appointment with an endocrine surgeon on May 2019 in Italy. A physical examination performed following postoperative recovery revealed recent surgical scar at the base of the neck near the manubrium, the absence of apparent neck lymph nodes, and non-palpable thyroid gland. The patient was tall (1.80 m), with a long neck. Based on the result of nasopharyngeal laryngoscopy, abnormalities were not observed. US re-examination and computed tomography scan revealed the following findings: presence of the thyroid gland and nodule (5 × 6 mm) in the left lobe and hypoechoic nodule (~4 × 4 mm) in the thyroid’s right lobe (Supplementary Figure 2). Thyroid function was normal. Previous clinical documentation was translated into Italian and reviewed. The foreign operative procedure demonstrated supposed thyroidectomy and left latero-cervical lymphadenectomy (levels III, IV, and V). Central compartment lymphadenectomy (levels VI and VII) was not observed. Histological examinations were reviewed, confirming the absence of the thyroid gland and the presence of normal thymic tissue and PTC lymph node metastasis (Supplementary Figure 1). The patient was scheduled for thyroidectomy with central compartment neck dissection (levels VI and VII) with neural monitoring. The intraoperative finding showed a normal volume thyroid gland, left suspicious nodule (<10 mm), and tenacious adhesions between the gland and strap muscles from previous surgery. The thyroid gland was positioned high in the neck, due to the patient’s long neck. Patient’s postoperative course was regular. Post-operative laryngeal examination revealed normal motility of the vocal cords. Histological examination revealed left lobe sclerosing variant PTC (5 mm) and 1/8 micrometastases of the central compartment lymph nodes (Supplementary Figure 1). BRAF V600E gene mutation was positive. After 4 weeks, the patient received radioiodine ablation therapy with 200 mCi of I-131.

**Reference Case 2: wrong-side lobectomy**

A 31-year old Sinhalese woman was referred on March 2016 because of right Bethesda III, 10-mm thyroid nodule. She underwent left lobectomy by mini-incision (3 cm). The pathology revealed normal thyroid tissue. On April 2016, she underwent right thyroid resection. The final pathology indicated right benign follicular adenoma. Patient’s recovery after two surgeries was functional, and the patient was re-examined regularly. Supplementary Figure 3 describes the internal audit performed for the case described above.

**Reference Case 3: lymph node excision instead of thyroidectomy**

In June 2013 a 21-year old Italian woman affected by a 0,8 cm papillary carcinoma of the right thyroid lobe with central and right lateral compartment lymph nodes metastasis was referred to a low-volume endocrine surgery unit and scheduled for surgery. Planned procedure was a total thyroidectomy with central and right lateral compartment clearance. Final hystological diagnosis reported bilateral papillary carcinoma substituting the entire lobes with huge lymphatic infiltration as severe thyroiditis and right lateral lymph nodes metastasis. The patient presented a post-operative left vocal cord palsy. The WSS was revealed in September 2013 when, during a routine follow-up US scan before radioiodine ablation, regular thyroid gland was visualized with suspicious 0.8cm nodule of the right side and suspicious lymph nodes of central and left lateral compartment.. Tireoglobulin level had always been elevated after surgery. Hystological examination was revised and thyroid lobes turned out to be just metastatic lymph nodes of the central compartment. Thyroid tissue was not present in the specimen. In October 2013 the patient was referred to our center. US scan performed in our department confirmed the presence of the thyroid gland and of the suspicious lymph nodes in both central and left lateral compartment. Left vocal cord palsy was still present so, hoping it was transient and not permanent, to avoid the risk of a bilateral palsy, we decided to postpone surgery in January 2014. Unfortunately in January the palsy was still present but waiting any longer was not ethical and potentiallly harmful so the patient was scheduled for total thyroidectomy with central and left lateral compartment clearance. Post-operative course was uneventful and the patient was discharged in third post-operative day. Final hystological examination revealed bilateral papillary carcinoma with central and left lateral compartment lymph nodes metastasis. After two cicles of radioiodine ablation at high dose the patient is disease free. Left vocal cord palsy is permanent.

**Reference Case 4: thymectomy instead of thyroidectomy**

A 41-year old Caucasian woman was referred to surgery in South Italy (Sicily) on January 2002 because of toxic goitre. Total thyroidectomy was planned. However, during surgical exploration, some difficulties were described in thyroid gland detection, driving the surgeon to invoke a probable thyroid agenesis, after the removal of some fatty and thymic tissues. Postoperative complications were not observed. Final histological examination reported thymic tissue. Thereafter, the patient continued to complain compressive symptoms and experienced anxiety and tachycardia. Subsequently, a neck US was performed, and a multinodular goitre was confirmed. Based on hormonal evaluation, decreased thyroid-stimulating hormone level with normal tri-iodothyronine and thyroxine levels was observed. The patient was referred to our institution for a surgical consultation. Preoperative laryngoscopy revealed normal results, with preoperative parathyroid hormone (PTH) level within the normal range. Total thyroidectomy was planned and performed on 21 May 2002. The thyroid gland was positioned high in the neck, considering the patient’s long neck in this case. No scar tissue was found at the level of the thyroid bed. Post-operatively, normal vocal fold motility and normal PTH level were observed. The patient was discharged on second postoperative day. Final pathological report confirmed a multinodular hyperfunctional goitre.

**Reference Case 5: thymectomy instead of thyroidectomy**

A 17-year-old Caucasian woman underwent surgical consultation because of a single, Bethesda V, 15-mm right lobe nodule. Thyroid cancer was ruled out in this patient. Total thyroidectomy was planned on 12 July 2019. In the surgical report, surgical steps for total thyroidectomy were described, without mentioning any difficulties in thyroid gland detection. The postoperative course was uneventful. The patient was discharged on second postoperative day. Based on the final pathological report, a surgical specimen sent as thyroid gland (right lobe, 6 × 1.5 × 0.8 cm; isthmus, 0.5 cm; left lobe, 4.5 × 1.5 × 0.6 cm) with some lymph nodes adherent to the upper pole of the supposed thyroid gland was observed. According to the microscopic pathological diagnosis, any thyroid tissue was not observed, and ‘ectopic’ normal thymic tissue (8.3 g) with 5 PTC metastatic lymph nodes was described.

After acquiring the final histological examination, the patient consulted an endocrine surgeon. The surgeon-performed neck US revealed a normal thyroid gland with a 15-mm right lobe nodule (preoperative cytology Bethesda V) adherent to the thyroid capsule and a suspicious level III right lateral neck node. The patient’s parents refused the lateral neck node US-FNA proposed to preoperatively define the extent of surgical procedure. Preoperative laryngoscopy revealed normal results, with preoperative PTH level within the normal range. Total thyroidectomy and central (level VI) and lateral (levels II–V) neck dissection (the frozen section examination of the suspicious level III lateral neck node diagnosed as PTC metastatic lateral neck node) were performed on 25 July 2019. Postoperative complications were not observed. The patient was discharged on second postoperative day. Final pathological examination revealed a 15-mm angioinvasive follicular variant PTC in the right thyroid lobe with focal invasion of the periglandular fatty tissue. Invasion of the sternothyroid muscle resected en bloc with the thyroid was not observed. PTC metastases were observed in four out of the 31 resected lateral neck nodes. PTC metastases were not observed in the 9 retrieved central neck nodes.
